# Etiopathogenic role of ERK5 signaling in sarcoma: prognostic and therapeutic implications

**DOI:** 10.1038/s12276-023-01008-x

**Published:** 2023-06-19

**Authors:** Adrián Sánchez-Fdez, Sofía Matilla-Almazán, Sofía Del Carmen, Mar Abad, Elena Arconada-Luque, Jaime Jiménez-Suárez, Luis Miguel Chinchilla-Tábora, Mª José Ruíz-Hidalgo, Ricardo Sánchez-Prieto, Atanasio Pandiella, Azucena Esparís-Ogando

**Affiliations:** 1 Instituto de Investigación Biomédica de Salamanca (IBSAL), Salamanca, Spain; 2 Instituto de Biología Molecular y Celular del Cáncer (IBMCC)-CSIC, Salamanca, Spain; 3 Centro de Investigación Biomédica en Red de Cáncer (CIBERONC), CSIC-Universidad de Salamanca, Salamanca, Spain; 4 Moores Cancer Center, University of California San Diego, La Jolla, CA USA; 5 Departmento de Patología, Hospital Universitario de Salamanca, Universidad de Salamanca, Salamanca, Spain; 6 Universidad de Castilla-La Mancha, Laboratorio de Oncología Molecular, Unidad de Medicina Molecular, Centro Regional de Investigaciones Biomédicas, Unidad Asociada de Biomedicina UCLM, Unidad asociada al CSIC, Albacete, Spain; 7 Universidad de Castilla-La Mancha, Departamento de Química Inorgánica, Orgánica y Bioquímica, Área de Bioquímica y Biología Molecular. Facultad de Medicina, Albacete, Spain; 8 Universidad de Castilla-La Mancha, Departamento de Ciencias Médicas, Facultad de Medicina, Albacete, Spain; 9 Departamento de Biología del Cáncer, Instituto de Investigaciones Biomédicas ‘Alberto Sols’ (CSIC-UAM), Unidad Asociada de Biomedicina UCLM, Unidad Asociada al CSIC, Madrid, Spain; 10 Instituto de Investigaciones Biomédicas ‘Alberto Sols’, Consejo Superior de Investigaciones Científicas (IIBM-CSIC)-Universidad de Castilla-La Mancha (UCLM), Albacete, Spain

**Keywords:** Sarcoma, Tumour biomarkers, Cancer models, Targeted therapies, Translational research

## Abstract

Sarcomas constitute a heterogeneous group of rare and difficult-to-treat tumors that can affect people of all ages, representing one of the most common forms of cancer in childhood and adolescence. Little is known about the molecular entities involved in sarcomagenesis. Therefore, the identification of processes that lead to the development of the disease may uncover novel therapeutic opportunities. Here, we show that the MEK5/ERK5 signaling pathway plays a critical role in the pathogenesis of sarcomas. By developing a mouse model engineered to express a constitutively active form of MEK5, we demonstrate that the exclusive activation of the MEK5/ERK5 pathway can promote sarcomagenesis. Histopathological analyses identified these tumors as undifferentiated pleomorphic sarcomas. Bioinformatic studies revealed that sarcomas are the tumors in which ERK5 is most frequently amplified and overexpressed. Moreover, analysis of the impact of ERK5 protein expression on overall survival in patients diagnosed with different sarcoma types in our local hospital showed a 5-fold decrease in median survival in patients with elevated ERK5 expression compared with those with low expression. Pharmacological and genetic studies revealed that targeting the MEK5/ERK5 pathway drastically affects the proliferation of human sarcoma cells and tumor growth. Interestingly, sarcoma cells with knockout of *ERK5* or *MEK5* were unable to form tumors when engrafted into mice. Taken together, our results reveal a role of the MEK5/ERK5 pathway in sarcomagenesis and open a new scenario to be considered in the treatment of patients with sarcoma in which the ERK5 pathway is pathophysiologically involved.

## Cancer: Signaling pathway offers new drug target for sarcoma

Therapies directed at a key signaling pathway could help reduce the growth of sarcomas, connective tissue cancers that affect fat, muscles, nerves and other tissues. Azucena Esparís-Ogando from the Instituto de Investigación Biomédica de Salamanca (IBSAL), Spain, and colleagues showed that in a genetically engineered mouse model the activation of the MEK5 enzyme activates the ERK5 enzyme, a regulator of genes involved in cell proliferation, differentiation and survival, and promotes the development of soft-tissue sarcomas. Drugs that block MEK5 or ERK5 activity reduced the growth of sarcoma cell lines and sarcoma tumors implanted into mice. A similar drug strategy could be applicable in cancer treatment in humans given the authors’ demonstration that ERK5 is frequently overexpressed in human sarcomas and that patients with elevated ERK5 have worse survival outcomes.

## Introduction

Mitogen-activated protein kinases (MAPKs) are a family of proteins that participate in several physiological processes, including proliferation, differentiation, cell death and stress sensing^1^. In mammals, four major pathways that use these proteins have been described, namely, the ERK1/2, ERK5, JNK and p38 MAPK pathways.

MAPK pathways require a cascade of kinase phosphorylations that finally lead to activation of the MAPK^2–4^. ERK5 is activated by the upstream kinase MEK5, which in turn may be activated by the kinases MEKK2 or MEKK3^3^. The activation of MEK5 and ERK5 requires the phosphorylation of two amino acid residues in a region called the activation loop^5,6^. In MEK5, those residues are Ser^311^ and Thr^315^. In fact, phosphomimetic mutations in MEK5 in which those residues are substituted by aspartic acid generate a constitutively active form of MEK5^7^. Once activated by phosphorylation at those sites, MEK5, which is endowed with threonine and tyrosine kinase activities, can in turn act on the TEY motif of ERK5, phosphorylating Thr^218^ and Tyr ^220^ ^8–10^ residues. Such phosphorylation activates ERK5, which is then capable of substrate phosphorylation and autophosphorylation^11,12^. In fact, upon activation at the TEY microdomain, ERK5 can phosphorylate several residues located in its C-terminal tail that are important in the regulation of its transcriptional activity^11^.

The findings of various studies support a role of the ERK5 pathway in the control of cell proliferation^3,4,8,9,13–18^. In fact, the embryonic lethality elicited by deletion of *ERK5* in mice was attributed to reduced proliferation of vascular endothelial cells necessary for the formation of blood vessels^19–21^. In addition, other studies mainly carried out in tumor cell lines have shown that a reduction in the levels of ERK5 or its pharmacological targeting decreased the proliferation of a number of cell lines or even caused cell death, raising the possibility that targeting of that pathway could be used for therapeutic purposes^3^. However, while the evidence for a role of the ERK5 pathway in cell proliferation suggests that it could play a pathophysiological role in tumor development, evidence for an etiopathogenic role of this pathway in cancer has only recently been demonstrated^7^. That evidence has been obtained by generating mice expressing a constitutively active form of MEK5, named MEK5DD, that was engineered to mimic the phosphorylation of Ser^311^ and Thr^315^ residues of MEK5^7^. Expression of MEK5DD in mice resulted in the development of lung adenocarcinomas in 46% of the animals, which also expressed constitutively activated ERK5^7^. These studies demonstrated that the mere targeting could be exploited therapeutically. In fact, genetic or pharmacological inhibition of the ERK5 pathway in human lung cancer cells resulted in substantial inhibition of their proliferation in vitro and tumorigenicity in vivo^22^.

In addition to the promotion of lung adenocarcinoma formation by the expression of MEK5DD in mice, almost 10% of the animals also developed subcutaneous tumors. In the present report, we demonstrate that these tumor lesions are sarcomas. Among rare diseases, sarcomas constitute a large heterogeneous group of tumors of mesenchymal origin that can arise at any anatomical site within either bone or soft tissue^23^. The vast majority of sarcomas (~80%) are soft tissue sarcomas (STSs)^24^. They are classified and named according to the normal tissue that the tumor most closely resembles^25^. The World Health Organization (WHO) recognizes >100 histologic subtypes. As a rare disease, sarcoma is diagnosed in only four to five per one hundred thousand people annually^26^. Although sarcomas can develop at any age, many of those affected are children, adolescents, or young adults. Sarcomas account for 1% or less of all adult cancers and for ~10% of pediatric tumors.

Traditionally, surgery and radiation therapy are the main treatment strategies for sarcomas confined to the single area of tissue in which they originate. Chemotherapy is often used when a sarcoma has already spread. One of the major medical objectives in the sarcoma field is the finding of novel therapies that, by targeting essential molecular components involved in the pathophysiology of the disease, may result in therapeutic benefit. This is especially relevant in those cases in which currently used therapies fail to control the disease. The fact that constitutive activation of the MEK5/ERK5 pathway resulted in sarcomagenesis in mice led us to explore the relevance of that pathway in that disease, especially considering the possibility of its therapeutically targeting. We report here that sarcomas are the human cancer type in which ERK5 is most frequently amplified and overexpressed. Moreover, the results of genetic as well as pharmacological studies suggest that targeting this pathway drastically affects the proliferation of human sarcoma cells. Together, the results reported here open a new therapeutic scenario for the treatment of patients with sarcoma, in which the ERK5 pathway may play a pathophysiological role.

## Materials and Methods

### Reagents and antibodies

BIX02189 was purchased from Selleckchem (Houston, TX, USA), and JWG-071 was purchased from MedChemExpress (NJ, USA). Protein A and Gammabind G-Sepharose were obtained from GE Healthcare (Uppsala, Sweden). Neuregulin was obtained from Prospec Protein Specialists (Rehovot, Israel). Puromycin, polybrene and MTT were purchased from Sigma‒Aldrich (St. Louis, MO, USA). Other labware and standard chemicals were obtained from BD Biosciences (San Jose, CA, USA), Sigma‒Aldrich (St. Louis, MO, USA) and Merck (Darmstadt, Germany).

The anti-ERK5 antibodies utilized in this study have been previously developed and described^7,8,27^. The other antibodies were purchased from the following manufacturers: anti-MEK5, from Enzo Life Sciences (Farmingdale, NY, USA); anti-Flag and anti-tubulin, from Sigma‒Aldrich (St. Louis, MO, USA); anti-Calnexin, from Stressgen Bioreagents (Victoria, BC, Canada); anti-vimentin clone V9, anti- S100 clone EP32, anti-actin clone HHF35, anti-desmin clone DE-R-11, and anti-CD34 clone QBEnd/10, from Leica Biosystems (Newcastle, UK); anti-caldesmon clone TD107, from Vision BioSystems Novocastra (Newcastle, UK); anti-Ki67 clone SP6, from Vitro Master Diagnostica (Granada, Spain); and anti-cleaved caspase-3 (Asp175), from Cell Signaling (Danvers, MA, USA). Horseradish peroxidase-conjugated secondary antibodies were obtained from Bio-Rad (Hercules, CA, USA).

### Cell culture

The GCT, SKLMS1, SKUT1, SJCRH30, and sNF96.2 sarcoma cell lines were purchased from ATCC (American Type Culture Collection). After thawing and expansion, 10 vials of each were frozen. Upon thawing, they were maintained in culture for no longer than 2 months. HeLa cells were also obtained from ATCC.

Cells were cultured in high-glucose (4.5 g/L) DMEM (Dulbecco’s modified Eagle’s medium) containing L-glutamine (4 mM) and L-pyruvate (5 mM) or in RPMI 1640 medium (SJCRH30). The complete growth medium was made by adding 10% FBS (fetal bovine serum) and antibiotics (penicillin (100 U/mL), streptomycin (100 µg/mL)). Cell culture reagents were purchased from Thermo Fisher (Madrid, Spain). All cell lines were cultured at 37 °C in a humidified atmosphere in the presence of 5% CO_2_ and 95% air. Mycoplasma testing was routinely performed.

### Western blotting, CRISPR/cas9, lentivirus-mediated knockdown, cell proliferation assay and clonogenic assay

The methods used for cell lysis, measurement of protein concentrations in cell lysates, immunoprecipitation and Western blotting have been previously described^28–30^.

Generation of *ERK5* and *MEK5* knockout clones by CRISPR/Cas9, as well as production of lentiviruses and infection of the sarcoma cell lines with TRC lentiviral pLKO vectors containing human *ERK5* and *MEK5* shRNA sequences, were performed as previously described^22^. *ERK5* or *MEK5* was knocked out in the target cells using a CRISPR/Cas9 system (references sc-400891 and sc-400891-HDR for ERK5 and sc-401688 and sc-401688-HDR for MEK5) and a control CRISPR/Cas9 plasmid (reference sc-418922) from Santa Cruz Biotechnology.

Cell proliferation assays were carried out in 24- or 6-well plates. To analyze the antiproliferative effects of BIX02189 and JWG-071, the appropriate concentrations of these inhibitors were added to the medium. Proliferation was quantified by an MTT-based assay as previously described^28,29^ or cell counting^22^. The values were always normalized to those in control cells (sh Control cells for shRNA knockdown experiments, Scramble cells for CRISPR knockout experiments, and vehicle or untreated cells for pharmacological experiments) and presented as the mean ± SD of two or three independent experiments with three or four biological replicates each.

Clonogenic assays were performed by seeding GCT (1,600 cells/well), SKLMS1 (800 cells/well), SKUT1 (400 cells/well), and SJCRH30 (1,000 cells/well) cells in 6-well plates and culturing the cells for 13 days. The cells were then fixed and stained with crystal violet, and colonies were counted, excluding those <5 mm in diameter, by using ImageJ software.

### MEK5DD transgenic mice and xenograft models

Site-directed mutagenesis of the relevant Ser^311^ and Thr^315^ residues of human MEK5 (UniProtKB accession number Q13163) to aspartic acid (MEK5DD) was performed using the QuikChange II Site-Directed Mutagenesis Kit (Stratagene, Madrid, Spain) following the provider’s instructions. The general procedures used for the preparation of the MMTV-Flag-MEK5DD construct and its microinjection into fertilized eggs of FVB mice for the generation of MEK5DD transgenic mice have been previously described^7^. Transgenic offspring were identified by PCR analysis of genomic DNA from tail snips with specific primers corresponding to MEK5DD (Forward: 5'-GGTGAATGACATAGCCAAGGA-3', Reverse: 5'-ATTGAACTGCACGATGAACG-3') and MMTV (Forward: 5'-CCCCTTTCGTGAAAGACTCG-3', Reverse: 5'-CCCCTCCTTGGTATGGAAAA-3').

Mice on a pure FVB genetic background were housed in a pathogen-free environment and were handled by authorized personnel at the animal facility according to legal and institutional requirements. (Authorization number 124). After the detection of any tumor, mice were humanely sacrificed, and the resected tumors were divided into two pieces. One piece was utilized for biochemical analyses, while the other was formalin-fixed, paraffin-embedded, sectioned, and stained with H&E for histochemical analyses. Immunohistochemistry was performed using the Bond Polymer Refine Detection Kit (Leica Biosystems, Newcastle, UK).

For the in vivo pharmacological studies testing the antitumoral efficacy of JWG-071, 16 female BALB/c nude mice (Charles River, Wilmington, MA, USA) were randomly divided into 2 groups of 8 mice each. 8×10^6^ GCT cells were injected following standard procedures into the left and right flanks of the mice in one group (2 tumors per mouse), and 6×10^6^ SJCRH30 cells were injected into both flanks of the mice in the other group. After engraftment, the mice in the GCT and SJCRH30 groups were further divided into 2 subgroups each (4 mice per group), with a similar mean weight and mean tumor volume (~150 mm^3^) for the mice in each subgroup. Thereafter, the mice in one GCT subgroup and one SJCRH30 subgroup received daily i.p. treatment with JWG-071 (30 mg/kg dissolved in 30% 2-hydroxypropyl beta-cyclodextrin), while the mice in the control subgroups received vehicle (30% 2-hydroxypropyl beta-cyclodextrin). Tumors were measured every 2 days using a digital caliper until the end of the experiment. Tumor volume was calculated by the following formula: *V(mm*^*3*^*)* *=* *L* *x* *W*^*2*^ *x* *0.5*. Resected tumors were formalin-fixed, paraffin-embedded and sliced into 3-µm sections. Ki67 (dilution 1:100) and cleaved caspase-3 (dilution 1:250) immunoreactivity was automatically assessed in the Leica Bond-III stainer by using the Bond Polymer Refine Detection Kit (Leica Biosystems, Newcastle, UK). For antigen retrieval, Bond Epitope Retrieval Solution 2 (pH 9) was used. Quantification of Ki67- or cleaved caspase-3-positive cells was performed with ImageJ software.

Additionally, female BALB/c nude mice were used to determine the effect of *MEK5* or *ERK5* knockout on tumorigenesis in vivo. Mice were implanted with 2 × 10^6^ or 4 × 10^6^ GCT cells (Sc, ERK5 KO or MEK5 KO cells; *n* = 2 tumors for each cell model) to establish xenografts. Tumor growth was periodically measured until the end of the experiment.

### Patients

All procedures with human samples were carried out in accordance with the guidelines of the Declaration of Helsinki on ethical principles for medical research involving human subjects. Patients provided written informed consent for the usage of the samples. Approval for such use was obtained from the Institutional Review Board Ethics Committee on Human Research of the hospital. A total of 59 frozen human tumor samples and 10 normal tissue samples were obtained from patients at the University Hospital of Salamanca. Specifically, the tumor samples were obtained from patients diagnosed with leiomyoma (*n* = 11), leiomyosarcoma (*n* = 12), lipoma (*n* = 5), liposarcoma (*n* = 12), malignant peripheral nerve sheath tumor (MPNST) (*n* = 4), schwannoma (*n* = 1), rhabdomyosarcoma (*n* = 4), and undifferentiated pleomorphic sarcoma (UPS) (*n* = 10). Some normal tissue samples corresponded to the counterpart of a tumor sample from the same patient.

Homogenization of the human tissue samples, immunoprecipitation and Western blot analysis were performed as previously described^7,22^. Quantification of the patients’ ERK5 levels was performed by analyzing ERK5 immunoblot bands with Image Lab Software 6.0 (Bio‐Rad Laboratories). For normalization of the ERK5 signal, an internal control was used. Boxplot representations of all patient samples (*n* = 69) and Kaplan‒Meier survival curves for those patients with available survival data (*n* = 55) were generated using GraphPad Prism 5.0 software (La Jolla, CA, USA). The threshold for dividing the patients into the low/high ERK5 expression cohorts was based on the separation between the first tertile (low) and the other two tertiles (high).

### In silico *studies*

The evaluation of *MEK5* and *ERK5* molecular alterations across human tumors was carried out by accessing the complete list of TCGA PanCancer Atlas studies collected in the cBioPortal bioinformatic tool (http://cbioportal.org)^31^. This list includes a total of 32 individual studies involving 10967 tumor samples from *n* = 10953 patients, where copy number alterations (CNAs) as well as mutations were analyzed. In addition, the mRNA expression level of ERK5 and its correlation with CNA was explored in the aforementioned TCGA studies. Putative copy number calls were made using GISTIC 2.0, and mRNA expression levels were determined by RNA Seq V2 RSEM (mRNA expression z scores relative to those in diploid samples).

To further analyze the *MEK5* and *ERK5* molecular alterations specifically present in sarcoma tumors, we selected the Sarcoma (TCGA, PanCancer Atlas) study (*n* = 251), where gene amplification, gene gain and mRNA upregulation can be differentially analyzed in several sarcoma subtypes. Furthermore, the potential existence of *ERK5* mutations in sarcoma was explored in greater depth in the Sarcoma (MSK, 2022) study, which contains targeted sequencing data of 2138 sarcoma samples.

On the other hand, the most frequent somatic focal copy number gain events in sarcoma patients were evaluated by accessing the Firebrowse database (http://www.firebrowse.org) and specifically selecting the Sarcoma cohort (*n* = 261), whose source data were obtained from the Broad Institute TCGA Genome Data Analysis Center^32^.

The copy number alteration events were identified by GISTIC and classified as deletion (log2(CN ratio) ≤ −1), loss (−1 < log2(CN ratio) < −0.42), gain (0.58 < log2(CN ratio) < 1.3), or amplification (log2(CN ratio) ≥ 1.3).

The Gene Expression Viewer tool available in Firebrowse was also used to validate the ERK5 mRNA expression levels across several tumor types. The *ERK5* differential expression plot was generated from RSEM (log2) data, including both tumor and normal samples. Similar analyses were performed with the Gene Expression Profiling Interactive Analysis (GEPIA2) database (http://gepia2.cancer-pku.cn/)^33^, where *MEK5* and *ERK5* gene expression profiles are quantified as transcripts per million (TPM) values and represented across all tumor samples and paired normal tissues.

### Statistical analyses

The associations between variables were analyzed for each in vitro cell proliferation assay with Fisher’s exact test, and comparisons of continuous variables between groups were carried out using two-tailed Student’s *t*-test. The Kolmogorov‒Smirnov test was used to determine whether the data from the in vivo tumor growth experiments followed a normal distribution. One‐way ANOVA followed by the Bonferroni correction for multiple comparisons was used to compare the control group with each treatment group. Differences between groups were considered significant if the *p*-value was ≤0.05 (*), ≤0.01 (**) or ≤0.001 (***). Statistical analyses were performed by using GraphPad Prism 5.0 software (La Jolla, USA). The significance of the differences in the overall survival of patients from the University Hospital of Salamanca was determined with GraphPad Prism 5.0 software by using the Gehan-Breslow-Wilcoxon test. In some cases, additional statistical information is indicated in the appropriate figure legend.

## Results

### Sarcomagenesis in mice expressing constitutively active MEK5

**Fig. 1:**
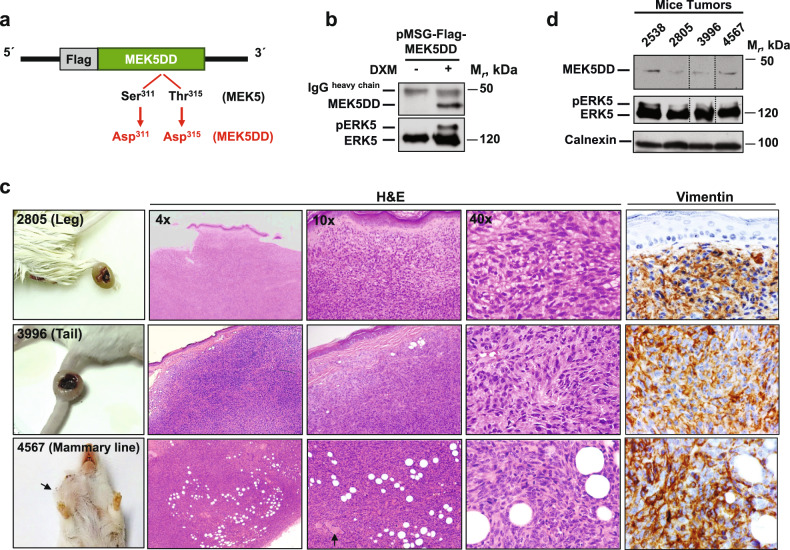
Transgenic mice with active MEK5 develop sarcomas. **a** Sites in MEK5 (Ser^311^ and Thr^315^) were mutated to aspartic acid to create the constitutively active form MEK5DD tagged with a Flag sequence. **b** pMSG-Flag-MEK5DD was transfected into HeLa cells. Cells were treated with or without 0.1 μM dexamethasone (Dxm). MEK5DD expression and its effect on ERK5 activation were assessed by SDS‒PAGE; MEK5DD and ERK5 were immunoprecipitated from cell extracts with anti-Flag or anti-ERK5 Pro1 antibodies, followed by Western blotting with anti-MEK5 or anti-ERK5 C-terminal antibodies, respectively. **c** Representative macroscopic images, hematoxylin-eosin and vimentin staining of tumors arising at different anatomical sites in MEK5DD transgenic mice. Atypical epithelioid cells with a haphazard arranged are shown in detail in 40x magnification images. Mammary ducts in the 4567 mouse are indicated by the black arrow. **d** Expression of MEK5DD (Flag) and ERK5 was assessed by Western blotting in tumors of four different transgenic mice after immunoprecipitation with the anti-Flag or anti-ERK5 Pro1 antibody followed by incubation with the same anti-Flag or anti-ERK5 C-terminal antibody. The dashed lines indicate lanes that were excised from the Western blot. Calnexin was used as a loading control.

The establishment of a MEK5DD transgenic model was previously described in detail^7^. In brief, we used a mutant form of MEK5 in which the relevant Ser^311^ and Thr^315^ residues, located in the MEK5 activation loop, were changed to aspartic acid (MEK5DD, Fig. 1a). As shown in Fig. 1b, expression of Flag-tagged MEK5DD in HeLa cells using the glucocorticoid-inducible vector pMSG caused a mobility shift of ERK5 (Fig. 1b), which is indicative of ERK5 activation^8,9^.

In vivo expression of the cDNA coding for Flag-tagged MEK5DD in FVB mice resulted in the development of lung adenocarcinomas^7^, as well as other types of tumors. Some of these other tumors formed in the soft tissue of different anatomical sites, such as the limbs, tail, mammary line, dorsum, or abdominal region (Fig. 1c and Supplementary Fig. 1). The incidence of these tumors was 9.7% in a total of 93 mice analyzed, and they were observed at ~14–16 months of age. Histopathological analysis of these masses allowed their identification as high-grade sarcomas. Morphologically, all tumors were composed of an admixture of atypical plump and spindle-shaped cells that were haphazardly arranged (Fig. 1c and Supplementary Fig. 1). In addition, the tumor cells in the tail were arranged in a vaguely storiform manner (tumor from mouse 3996, Fig. 1c) or exhibited entrapment of adipocytes and perineural invasion (tumor from mouse 4567, Supplementary Fig. 1) or mitotic figures (mouse 4012, Supplementary Fig. 1). In some cases, tumors ulcerated the epidermis (Fig. 1c) and entrapped muscular fibers (mouse 4031, Supplementary Fig. 1). The tumors that developed in the breast also showed evident mitotic figures (mouse 4567) and adipocyte or duct entrapment (mice 4567 and 4453, Fig. 1c and Supplementary Fig. 1, respectively). In the dorsum, mitotic figures and pleomorphic cells were evident (mouse 2538, Supplementary Fig. 1). Mouse 2805 developed a tumor mass in the hind limb composed mostly of atypical plump/epithelioid cells (Fig. 1c). Mouse 4808 developed a tumor within the abdominal area (Supplementary Fig. 1). Immunohistochemical analyses showed diffuse positivity of the tumor cells for vimentin, which excluded staining of epithelial tissue (Fig. 1c), indicating that they were lesions of mesenchymal origin. Staining for S100, actin, caldesmon, desmin, and CD34 was focal and nonspecific, indicating that the tumors could not be assigned to a particular differentiated sarcoma subtype. Globally, the findings of morphological and immunohistochemical analyses defined a phenotype compatible with undifferentiated pleomorphic sarcoma (UPS), formerly named malignant fibrous histiocytoma.

The tumors of these transgenic mice expressed Flag-tagged MEK5DD, as detected by Western blotting using an anti-Flag antibody, as well as substantial amounts of ERK5. Of note, an ERK5 band with retarded mobility, consistent with pERK5, was also detected in the analyzed tumors from the transgenic mice (Fig. 1d).

### ERK5 is frequently amplified and overexpressed in sarcoma

**Fig. 2:**
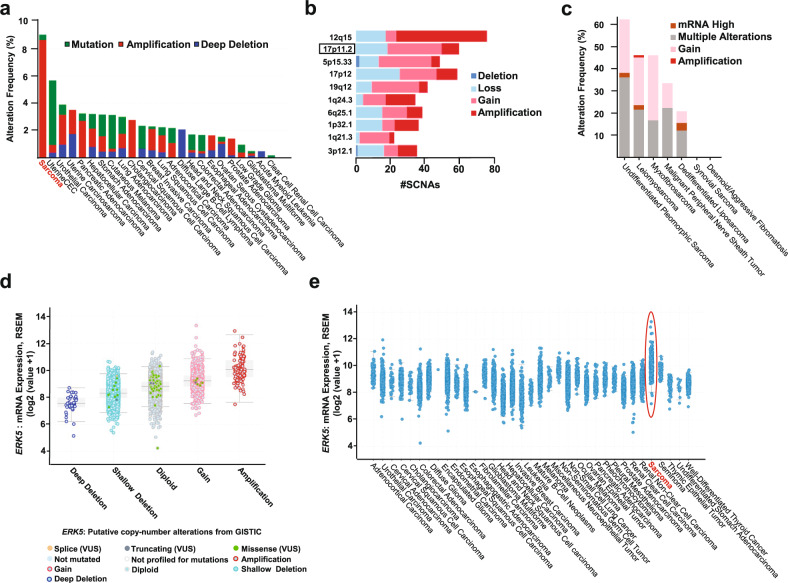
ERK5 is frequently amplified and overexpressed in sarcoma. **a** Molecular alterations in *ERK5* in different tumor types identified using the complete list of TCGA PanCancer Atlas studies (*n* = 10953 patients from 32 studies) available in cBioPortal. **b** The Firebrowse bioinformatics tool was used for the identification of focal CNAs in the Sarcoma cohort (*n* = 261 samples) from the Broad Institute TCGA Genome Data Analysis Center (2016). The top altered genomic locations were ranked and are presented from highest to lowest gain/amplification frequency. #sCNAs: number of somatic copy number alterations. **c** *ERK5* copy number gain/amplification and mRNA upregulation rates in patients with specific sarcoma subtypes from the TCGA dataset (*n* = 251 cases). **d** Correlation between *ERK5* copy number alterations (CNAs) and mRNA expression in sarcoma samples from the TCGA Firebrowse Legacy dataset. All samples were batch normalized. The copy number datasets were generated by the GISTIC algorithm. **e** *ERK5* mRNA levels in the different cancer types from the TCGA dataset.

The above data, which indicated a link between ERK5 pathway activation and sarcomagenesis in mice, led us to explore the potential presence of molecular alterations of the MEK5/ERK5 pathway in this disease. Analyses of molecular alterations of *ERK5* in different tumor types using the TCGA datasets (*n* = 10953 patients from 32 studies) available in cBioPortal (accessed March 25, 2022) showed sarcoma as the top-ranked cancer type, with molecular alterations in a total of 9.02% of cases (Fig. 2a). Amplifications accounted for 8.67%, while mutations accounted for only 0.39%. The rarity of mutations in *ERK5* in sarcoma was also indicated by analysis of the MSK2022 dataset (*n* = 2138 sarcoma samples, also accessed through cBioPortal), which showed no mutations in *ERK5*. Interestingly, data obtained from the Firebrowse online resource indicated that the second most altered cytogenetic region in sarcomas with respect to amplification/gain corresponded to 17p11.2 (Fig. 2b), which is the chromosomal region containing the *ERK5* locus. Analyses of specific sarcoma subtypes from the TCGA dataset showed that some of them did not show *ERK5* molecular alterations (synovial sarcoma (*n* = 10) and desmoid fibromatosis (*n* = 2)), while others presented high alteration frequencies (dedifferentiated liposarcoma (20.69%, *n* = 58)); malignant peripheral nerve sheath tumor (33.33%, *n* = 9); leiomyosarcoma (45.92%, *n* = 98); myxofibrosarcoma (45.83%, n = 24) (Fig. 2c). Of note, undifferentiated pleomorphic sarcoma was the subtype with the highest frequency of *ERK5* molecular alterations, found in 62% of the cases (*n* = 50). These analyses also showed a correlation between the type of copy number alteration and the levels of mRNA present in the sarcoma samples (Fig. 2d).

Transcriptomic analyses of mRNA levels in the tumors represented in the TCGA dataset revealed that sarcomas had the highest *ERK5* mRNA levels (Fig. 2e). This finding was also confirmed by analysis of data available in the Firebrowse and GEPIA2 databases. The Firebrowse data showed differences between normal and sarcomatous tumor tissue (median RSEM value for normal tissue: 8.65, median RSEM value for tumor tissue: 9.94; fold change: 2.45, Supplementary Fig. 2a). GEPIA2 reported expression values of 6.69 and 24.66 transcripts per million (TPM) for normal and sarcomatous tissues, respectively (Supplementary Fig. 2b).

In the TCGA combined study, and when analyzing *MEK5*, sarcoma was identified as the tumor type with the third-highest molecular alteration frequency, with gene alteration in 2.5% of the cases (Supplementary Fig. 3a). Transcriptomic data obtained from GEPIA2 confirmed that sarcomas were included among the tumor types in which the *MEK5* mRNA level was higher than that in the corresponding normal tissues (Supplementary Fig. 3b). The transcript values (TPM) for normal and tumor tissue were 12.64 and 15.38, respectively.

### Biochemical analysis of ERK5 expression in patient samples and its relationship to patient survival

**Fig. 3:**
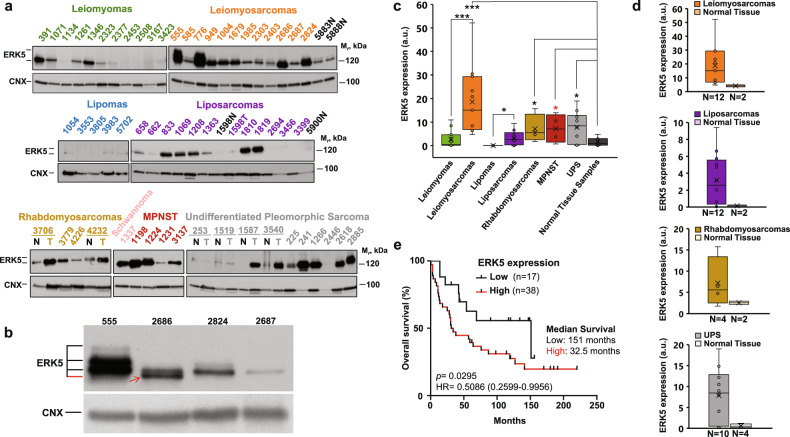
Biochemical analysis of ERK5 expression in patient samples and its relationship to patient survival. **a** ERK5 protein expression was analyzed in a cohort of patients from the University Hospital of Salamanca diagnosed with different sarcoma subtypes. One milligram of tumor protein was subjected to immunoprecipitation with the anti-ERK5 Pro1 antibody followed by Western blot analysis with the anti-ERK5 C-terminal antibody. The number above each lane indicates the tissue bank classification. N nontumor tissue, T tumor tissue. Calnexin was used as a loading control. **b** A total of 0.5 mg of tumor protein extract was subjected to immunoprecipitation and analysis as described above. The red arrow points to a possible truncated variant of ERK5. Calnexin was used as a loading control. **c** Box plot showing the expression level of ERK5 (a.u.) in 69 samples obtained from patients of the University Hospital of Salamanca. The Western blot shown in Fig. 3a was used for quantification of ERK5 band densities using ImageLab software. ERK5 signal expression was normalized to the signal expression of an internal sample used as a control. *p ≤ 0.05; ***p ≤ 0.001;  (red asterisk), *p* = 0.0539. **d** Box plots showing the ERK5 expression level (a.u.) in each subtype of tumor vs. the corresponding normal tissue. **e** Kaplan–Meier overall survival analysis of sarcoma patients from the University Hospital of Salamanca with available clinical data (*n* = 55) for 220 months of follow-up stratified by ERK5 protein expression. The lower tertile of expression was set as the cutoff threshold to sort patients into the low/high ERK5 expression groups.

We then explored ERK5 protein expression in human tumors from patients diagnosed with different sarcoma subtypes obtained from our University Hospital: leiomyoma (*n* = 11), leiomyosarcoma (*n* = 12), lipoma (*n* = 5), liposarcoma (*n* = 12), rhabdomyosarcoma (*n* = 4), schwannoma (*n* = 1), malignant peripheral nerve sheath tumor (*n* = 4) and undifferentiated pleomorphic sarcoma (*n* = 10). For some samples, paired normal tissue was available. ERK5 was immunoprecipitated with an anti-ERK5 antibody directed against the proline-rich 1 region of ERK5^27^, and Western blotting was performed with an antibody directed against the C-terminus of ERK5^8^. Internal controls were used to allow normalization of the signals. As shown in Fig. 3a, the amount of ERK5 varied between the samples. These Western blot analyses showed the presence of up to four different bands of ERK5 (Fig. 3b). Some of these bands were found to be coexpressed in individual samples, especially in those with high levels of ERK5 (e.g., sample 555, Fig. 3a, b). Of note, one of the ERK5 bands, detected in sample 2686, migrated at a higher rate than ERK5, suggesting that it might be a truncated variant (red arrow in Fig. 3b). Of the sarcoma types analyzed, all but lipoma exhibited higher ERK5 expression than that in the normal samples (Fig. 3c, d). Differences in ERK5 protein expression were also evident when comparing benign tumors (leiomyomas or lipomas) with their respective more aggressive forms (leiomyosarcomas or liposarcomas) (Fig. 3c). Analyses of the impact of ERK5 protein expression on patient survival in the patient cohort, which included patients with all sarcoma types analyzed with clinical follow-up data (*n* = 55), showed a significant negative relationship between ERK5 expression and patient survival (*p*-value = 0.0295, HR = 0.05086 (0.2599–0.9956)) (Fig. 3e). The median survival time of patients with high levels of ERK5 was 32 months, compared with 151 months for those with low expression levels, corresponding to a decrease of almost 5-fold in patient survival time.

### Functional relevance of the MEK5/ERK5 pathway in sarcoma

**Fig. 4:**
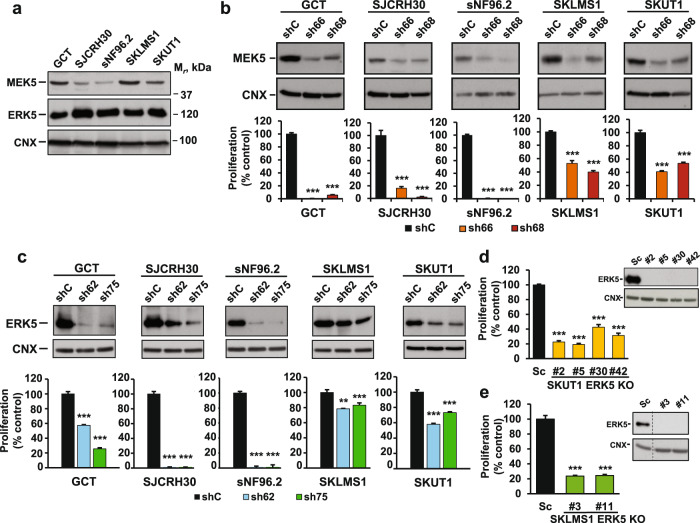
The MEK5/ERK5 pathway controls the proliferation of human sarcoma cell lines. **a** ERK5 expression was evaluated by immunoprecipitation of 1 mg of protein from sarcoma cell extracts with the anti-ERK5 Pro1 antibody followed by Western blotting with the anti-ERK5 C-terminal antibody. Seventy micrograms of protein extract was used to detect MEK5. Calnexin was used as a loading control. **b** ShRNA sequences sh66 and sh68 were used to decrease MEK5 protein levels in the different sarcoma cell lines, and MEK5 protein expression was compared with that in cells treated with shC (noncoding sequence) by Western blotting using 70 µg of cell extracts. The antiproliferative effect of the knockdown was analyzed by a cell counting assay when the control cells reached 85-90% confluence, and the proliferation rate is presented as a percentage of that in shC cells. Data are presented as the mean ± SD of two independent experiments with four biological replicates each. *p*-values: ***p ≤ 0.001. **c** Knockdown of ERK5 expression was carried out with shRNA sequences sh62 and sh75. ERK5 protein levels were analyzed by immunoprecipitation of 1 mg of protein with the anti-ERK5 Pro1 antibody followed by Western blotting with the anti-ERK5 C-terminal antibody. Calnexin was used as a loading control. The effect of ERK5 knockdown on cell proliferation was quantified by cell counting as described above, and the proliferation rate is presented as a percentage of that in shC cells. Data are presented as the mean ± SD of two independent experiments with four biological replicates each. *p*-values: **p ≤ 0.01; ***p ≤ 0.001. **d** SKUT1-ERK5 knockout clones (#2, #5, #30 and #42) were obtained by using CRISPR/Cas9 gene editing. The lack of ERK5 expression was determined by immunoprecipitation of 1 mg of protein extract followed by Western blotting with the anti-ERK5 antibody. Calnexin was used as a loading control. ERK5 KO clones and Sc cells were cultured for 5 days in 6-well plates, and the proliferation rate was measured by cell counting and is presented as a percentage of that in Sc cells. Data are presented as the mean ± SD of two independent experiments with four biological replicates each. *p*-values: ***p ≤ 0.001. **e** SKLMS1 ERK5 knockout clones (#3, #11) were also obtained. The lack of ERK5 expression was analyzed as described above. Cell counting was performed after 4 days of culture to study the effect on cell proliferation. Data are presented as the mean ± SD of two independent experiments with four biological replicates each. *p*-values: ***p ≤ 0.001.

The data obtained from transgenic mice, in silico analyses, and the protein patient expression biochemical data moved us to explore the importance of targeting the ERK5 pathway in sarcomas. To this end, we selected cell lines available from ATCC that express *MEK5* and *ERK5* mRNA, according to the Cancer Cell Line Encyclopedia: GCT (UPS/MFH), SJCRH30 (rhabdomyosarcoma), sNF96.2 (malignant peripheral nerve sheath tumor) and SKLMS1 and SKUT1 (leiomyosarcoma). Western blot analysis confirmed the presence of MEK5 and ERK5 in all the selected cell lines (Fig. 4a).

To explore the relevance of MEK5 and ERK5 to proliferation, the levels of these proteins were decreased in the different sarcoma cell lines by using lentivirus-mediated RNA interference, and the effects of such reductions in protein expression on cell proliferation were determined. Two shRNA sequences for each protein-coding gene were used, and the knockdown efficiency was assessed by Western blotting. MEK5 reduction translated into a decrease in proliferation in all the cell lines analyzed (Fig. 4b). In GCT, SJCRH30 and sNF96.2 cells, that effect was particularly drastic, indicating that MEK5 could be critical for the survival of those cells. After the same genetic approach, the decrease in the ERK5 levels resulting from RNA interference also showed a pronounced antiproliferative effect, with SJCRH30, sNF96.2 and GCT cells being the most affected (Fig. 4c and Supplementary Fig. 4a). Genetic downregulation of ERK5 did not have a major impact on Annexin V staining in these cells (Supplementary Fig. 4b). The effect of ERK5 knockdown on SKUT1 and SKLMS1 cell proliferation was moderate, although statistically significant.

Because of the moderate efficacy of ERK5 knockdown in SKUT1 and SKLMS1 cells, we decided to use a different genetic approach to explore the relevance of ERK5 to proliferation in these cells. To this end, CRISPR/Cas9 was used to generate SKUT1 and SKLMS1 cell-derived clones devoid of ERK5 protein expression. The results of Western blot analysis of ERK5 expression in several isolated clones are shown in Fig. 4d, e, as are their corresponding proliferation rates with respect to the Scramble control cells. These studies demonstrated that deletion of ERK5 profoundly compromised the proliferation of SKUT1 and SKLMS1 clones lacking ERK5 (Fig. 4d, e, respectively). GCT cells lacking ERK5 or MEK5 protein expression were also generated, and studies on their proliferation rate confirmed that a lack of either protein resulted in a substantial decrease in proliferation (Supplementary Fig. 4c and 4d). We also explored the tumorigenic potential of GCT cells and the corresponding MEK5 and ERK5 knockout clones in vivo. Cells with MEK5 knockout (clone #6) and ERK5 knockout (clone #45) were injected into mice, and the growth of tumors was compared to that in mice injected with the Scramble (Sc) control cells. As shown in Supplementary Fig. 4e, neither of the two clones lacking MEK5 or ERK5 was able to form tumors, while the Scramble control cells did form tumors that grew over time.

### Pharmacological targeting of the MEK5/ERK5 pathway in sarcoma

**Fig. 5:**
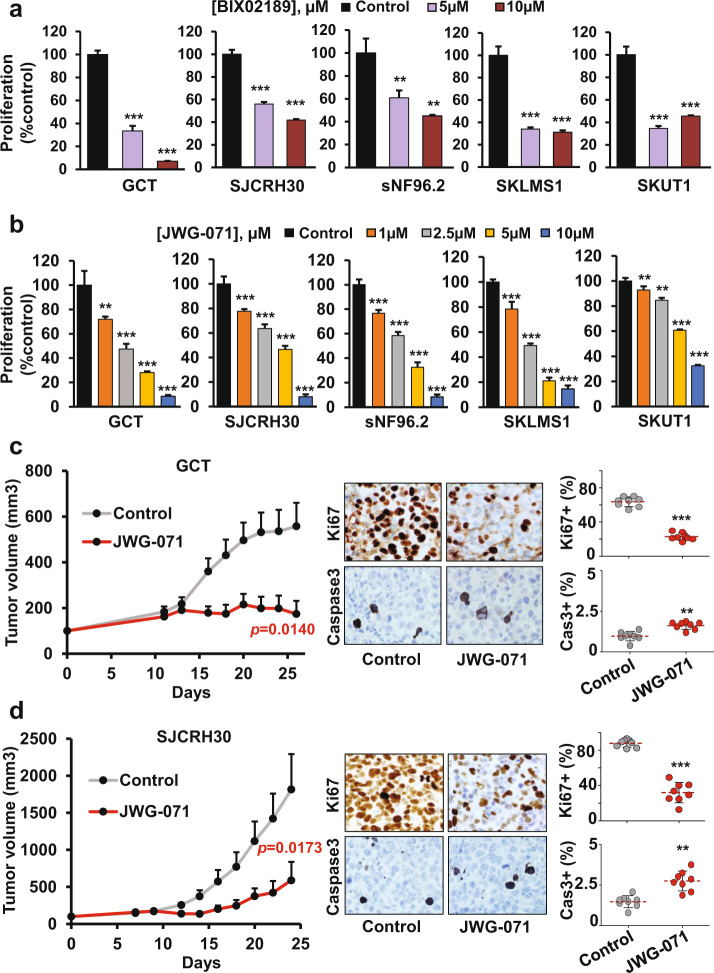
Pharmacological inhibition of MEK5 or ERK5 impairs the proliferation of sarcoma cell lines and tumor growth. Sarcoma cells were plated in 6-well dishes and, after 24 h, were treated with (**a**) BIX02189 or (**b**) JWG-071 at the indicated concentrations, and proliferation was quantified by cell counting after 4 or 3 days of treatment, respectively. The values were normalized to those in control cells (untreated) and are presented as the mean ± SD of two independent experiments with four biological replicates each. Statistical significance: **p ≤ 0.01; ***p ≤ 0.001. **c** Mice were implanted with GCT and (**d**) SJCRH30 cells. After engraftment, when the median volume of the tumors was ~150 mm3 (Day 11 for GCT or Day 9 for SJCRH30), the mice were treated with the ERK5 inhibitor JWG-071 or vehicle. Tumor progression was monitored every 2 days. Each point on the graph indicates the mean tumor volume ± SEM (left panels). Representative images of Ki67 and cleaved caspase-3 immunostaining in those tumors are shown at 40x magnification (middle panels). Expression was quantified in eight different images with ImageJ software and is presented as the percentage of positive cells relative to the total number of cells (right panels). The mean percentage (red dotted line) and p values are indicated: **p ≤ 0.01; ***p ≤ 0.001.

The above genetic studies aiming to analyze the functional relevance of this pathway in sarcoma were complemented with a pharmacological approach using MEK5 and ERK5 inhibitors. The effect of MEK5 inhibition on the growth of sarcoma cells was analyzed by using the inhibitor BIX02189. In vitro studies showed that this drug elicited an antiproliferative effect in all the analyzed sarcoma cell lines (Fig. 5a). Analogous studies using the ERK5 inhibitor JWG-071 showed that this drug resulted in a significant and dose-dependent reduction in cell proliferation in the five sarcoma cell lines analyzed (Fig. 5b). The effect of the drug was observed even at the lowest concentration used (1 μM). At the highest concentration used (10 μM), the effect was very pronounced in all the cell lines. The colony formation assay yielded similar findings (Supplementary Fig. 5a and 5b). Pharmacological inhibition of ERK5 did not induce a major increase in Annexin V staining (Supplementary Fig. 6).

To analyze whether targeting ERK5 with JWG-071 affects tumor growth in vivo, cells of two sarcoma cell lines (GCT and SJCRH30) were injected into the dorsal flanks of nude mice, and the action of the drug on the growth of the xenografts was monitored over time. Tumors formed from these injected cells were allowed to grow, and when the tumor volume was 150 mm^3^, the mice were randomized to receive vehicle or JWG-071. As shown in Fig. 5c, d, tumor growth in mice treated with JWG-071 was less robust than that in control mice treated with vehicle. Ki67 showed significantly lower immunostaining in treated compared with untreated mice, while no major difference was observed in the cleaved caspase-3 level (Fig. 5c, d).

## Discussion

MAPKs play essential roles in the control of several physiological processes, including cell proliferation. The finding that mice constitutively expressing active MEK5, which in turn causes activation of ERK5, developed lung adenocarcinomas demonstrated that the sole activation of that pathway was oncogenic^7^. In addition to developing lung tumors, a proportion of MEK5DD transgenic mice also developed soft tissue neoplasias, which we showed corresponded to sarcomas, particularly of the undifferentiated pleomorphic subtype. At present, the diagnosis of these undifferentiated sarcomas is a diagnosis of exclusion, in which other mesenchymal tumors are ruled out by IHC analysis of differentiation markers^34^. In sarcomatous tissue from MEK5DD transgenic mice, immunohistochemical staining failed to establish a line of differentiation, which led us, based also on morphological criteria, to classify these tumors as UPS. Considering that the incidence of that sarcoma subtype in the general population is 0.8-1 new cases per 100,000 per year, the 10,000-fold increase in incidence in our mouse model demonstrates that the activation of the ERK5 pathway is capable of promoting sarcomagenesis and opens the possibility of exploring the relevance of this pathway in human sarcomas. Sarcomas in MEK5DD mice appeared at ~14–16 months of age, which is the upper limit of middle age, equivalent to an age of ~50 years in humans^35^. In humans, UPS occurs most frequently in adults^34^. These characteristics indicate that activation of the MEK5/ERK5 pathway could be relevant to adulthood UPS.

The involvement of the MEK5/ERK5 pathway in the origin of tumors of different lineages, such as lung adenocarcinoma and sarcoma, in MEK5DD transgenic mice demonstrates that constitutive activation of this pathway may promote oncogenesis in distinct tissues. In fact, we previously reported the appearance of tumors in other locations in these MEK5DD mice, although with a lower frequency than lung adenocarcinomas or sarcomas^7^. Considering that the tumors that form in MEK5DD transgenic mice originate from different cellular sources, their common sensitivity to oncogenic transformation upon activation of the MEK5/ERK5 pathway indicates that they may share some molecular characteristics that favor the pro-oncogenic role of the MEK5/ERK5 pathway. It is also worth mentioning that MEK5/ERK5 signaling has been linked to tumors of diverse origins, such as breast carcinoma^12^, colon cancer^36^, prostate cancer^17^, hepatocellular carcinoma^37^, neuroblastoma^38^ and melanoma^39^, among others. In conclusion, the MEK5/ERK5 pathway may contribute to the etiology or pathophysiology of different types of tumors.

Several other lines of evidence suggest that the MEK5/ERK5 pathway may have a role in sarcomagenesis. For example, in a chemical carcinogenesis model of pleomorphic sarcoma with muscular differentiation, marked upregulation of ERK5 was observed, and ERK5 downregulation abrogated tumor growth^40^. Despite the differences between the sarcoma subtypes generated in the genetic and chemical carcinogenesis models, both studies demonstrate the contribution of the MEK5/ERK5 pathway to the pathophysiology of sarcoma. Indirectly, there are other examples that could suggest a mediating role of the MEK5/ERK5 pathway in soft tissue sarcomas. For example, rhabdomyosarcomas formation occurred in a genetically engineered mouse model aimed at Hedgehog signaling activation^41^. Notably, activation of the Hedgehog pathway was linked to MEK5/ERK5 activation^42^. The tyrosine kinase ALK has been shown to activate ERK5 in neuroblastoma, causing expression of the MYCN oncogene^38^. Interestingly, high levels of MYCN have been reported in alveolar rhabdomyosarcoma^43,44^ and synovial sarcoma^45,46^. Moreover, molecular alterations in ALK have been described in sarcomatous mesenchymal tumors^47–50^, including undifferentiated sarcomas^51^. Another scenario in which a role for ERK5 in sarcomas could be envisaged relates to the inflammatory cytokine IL-6. High levels of IL-6 were detected in the serum of soft tissue sarcoma patients and were associated with poor survival^52,53^. Interestingly, the requirement of ERK5 for IL-6 production in tumor cells has been reported^54^.

In silico studies demonstrated that molecular alterations in *ERK5* are most frequently detected in sarcomas. The main alterations found in sarcomas were amplifications of *ERK5*. Moreover, the chromosomal region containing the *ERK5* gene locus was among the most frequently amplified regions in sarcomas. Further studies aimed at delineating the transcriptomic profile of *ERK5* in different types of tumors also reported that *ERK5* expression was highest in sarcomas. Moreover, when molecular alterations and mRNA expression were considered collectively, up to half of the cases of undifferentiated pleomorphic sarcoma included either or both alterations. Biochemical analyses of ERK5 protein expression in different sarcoma subtypes showed a significant correlation between a high level of ERK5 protein expression and patient survival, suggesting that elevated ERK5 may favor tumor development. Together, these observations strongly suggest a pathophysiological role of ERK5 in human sarcomas.

Careful examination of the Western blots obtained with patient-derived samples showed that some sarcomas, particularly those with higher ERK5 expression, showed different ERK5 bands. While in the case of most tumors, the proteins corresponding to these additional ERK5 bands had higher molecular weights than the 120 kDa unphosphorylated ERK5, in some tumors (e.g., #2686), a protein with faster migration was detected. The molecular characteristics of the protein corresponding to that band are unknown, but in our experience in the analysis of tumor samples, such an ERK5 band is not common. The UniProt database contains four different ERK5 forms. Isoforms 3 and 4 contain C-terminal deletions incompatible with the molecular weight of the one detected in sample 2686. In fact, isoform 2 lacks the first 139 amino acids of isoform 1 (wild-type ERK5). Such an N-terminal deletion is expected to result in a form with even faster migration in SDS‒PAGE gels, and therefore, we do not think that it corresponds to the form detected in sample 2686. In the mouse, an isoform (mERK5b) that lacks the first 77 amino acids has been reported^55^. Additional work using antibodies directed against different epitopes of ERK5 as well as molecular analyses is needed to precisely define the alternative ERK5 forms expressed not only in sample 2686 but also in general in tumors. Those studies, combined with biological and functional analyses, may uncover novel roles of ERK5 in tumor pathophysiology.

The finding that mice expressing constitutively active MEK5 develop sarcomas, together with the results of in silico studies that pointed to sarcoma as the type of tumor in which ERK5 is most frequently altered, prompted preclinical studies aimed at determining whether modulation of the ERK5 pathway could be therapeutically relevant for that disease. Genetic loss-of-function studies of MEK5 and ERK5 using human sarcoma cell lines demonstrated that knocking down either of these proteins inhibited cell proliferation. Moreover, deletion of *ERK5* in sarcoma cell lines using CRISPR/Cas9 confirmed the relevant role of ERK5 in the proliferation of sarcoma cells. In addition to those genetic studies, pharmacological targeting of MEK5 or ERK5 by using BIX02189 or JWG-071, the respective kinase inhibitors of those proteins^56,57^, significantly decreased the proliferation of sarcoma cell lines. Moreover, in animal models bearing xenografts derived from sarcoma cells, the ERK5 inhibitor JWG-071 stopped (in the case of GCT) or slowed (in the case of SJCRH30) tumor growth. These data confirm that targeting the ERK5 pathway can inhibit the proliferation of human sarcomas.

In summary, the results reported in the present study demonstrate that the ERK5 pathway plays a relevant role in sarcoma. The finding that constitutive activation of that pathway causes sarcomagenesis in mice demonstrates that its deregulation may cause sarcomagenesis in humans. Moreover, the frequent molecular alterations and the transcriptomic upregulation of ERK5 in human sarcomas confirms the potential involvement of that pathway in the pathophysiology of that disease, at least in those cases in which such types of alterations are present. With respect to the potential relevance of targeting this pathway for therapeutic purposes, the genetic and pharmacological studies demonstrated that targeting MEK5 or ERK5 may decrease the proliferation of sarcoma cells and reduce tumor progression in vivo. Currently, the treatment approach for sarcomas combines surgery, radiation, and chemotherapy, but in recent years, immunotherapy and targeted therapy are moving this field toward precision medicine^58,59^. Our results suggest that MEK5/ERK5 targeting could be considered for sarcoma therapy. To date, several inhibitors of MEK5^56^ or ERK5^57,60–63^ have been developed. However, some of them have off-target effects, and others do not produce the antiproliferative effect achieved by *ERK5* genetic deletion^64^. More research is needed in this field to develop drugs directed against the MEK5/ERK5 pathway with the potential to enter clinical trials.

## Supplementary information

Supplementary Material
