# Supplementary material for: Etiopathogenic role of ERK5 signaling in sarcoma: prognostic and therapeutic implications
Source: Exp Mol Med. 2023 Jun 19;55(6):1247–57. doi: 10.1038/s12276-023-01008-x (PMC10317974; doi:10.1038/s12276-023-01008-x)
Supplement: Supplementary file 1 — Supplementary Material [file 12276_2023_1008_MOESM1_ESM.pdf]

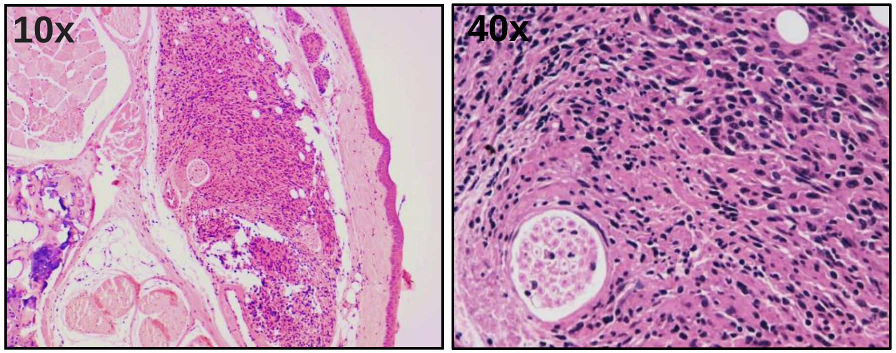

4567 Tail

Note entrapment of adipocytes and perineural invasion

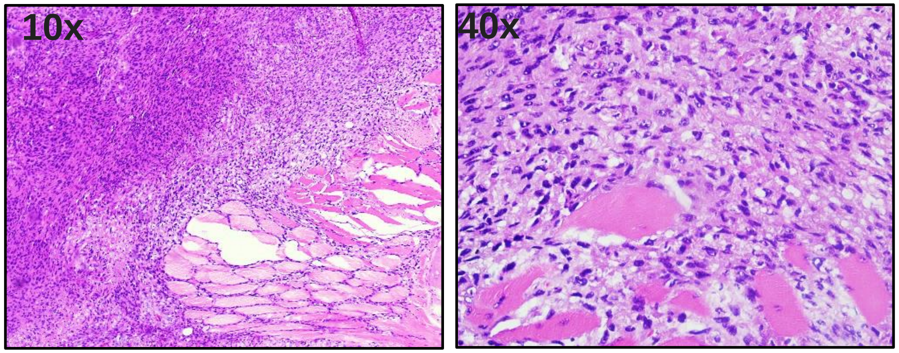

4031 Tail

Note entrapment of muscular fibers

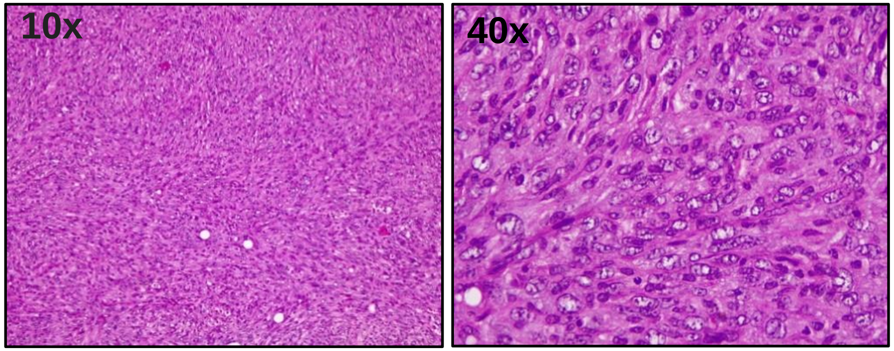

4012 Tail

Mitotic figures are evident

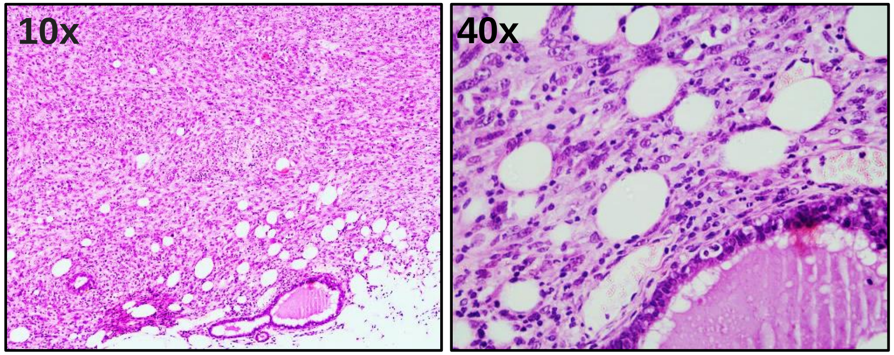

4453 Breast

Tumor entraps ducts and adipose tissue

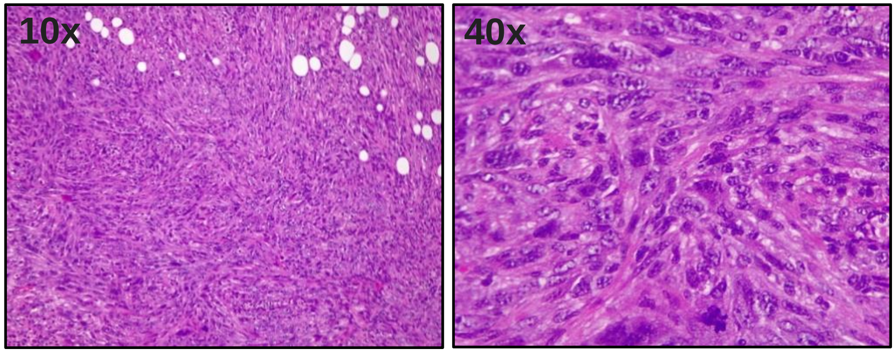

2538 Dorsum

Mitotic figures and pleomorphic cells are evident

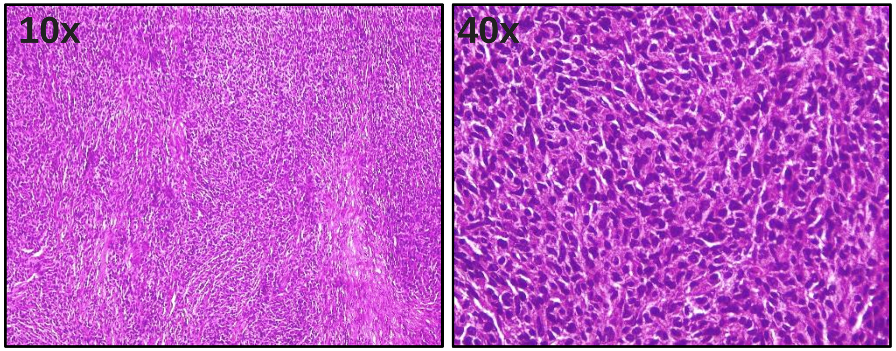

4808 Abdominal mass

Supplementary Fig. 1

Supplementary Fig. 1. Representative images of haematoxylin and eosin staining of tumor tissue from MEK5DD transgenic mice. Magnification: 10x and 40x.

a

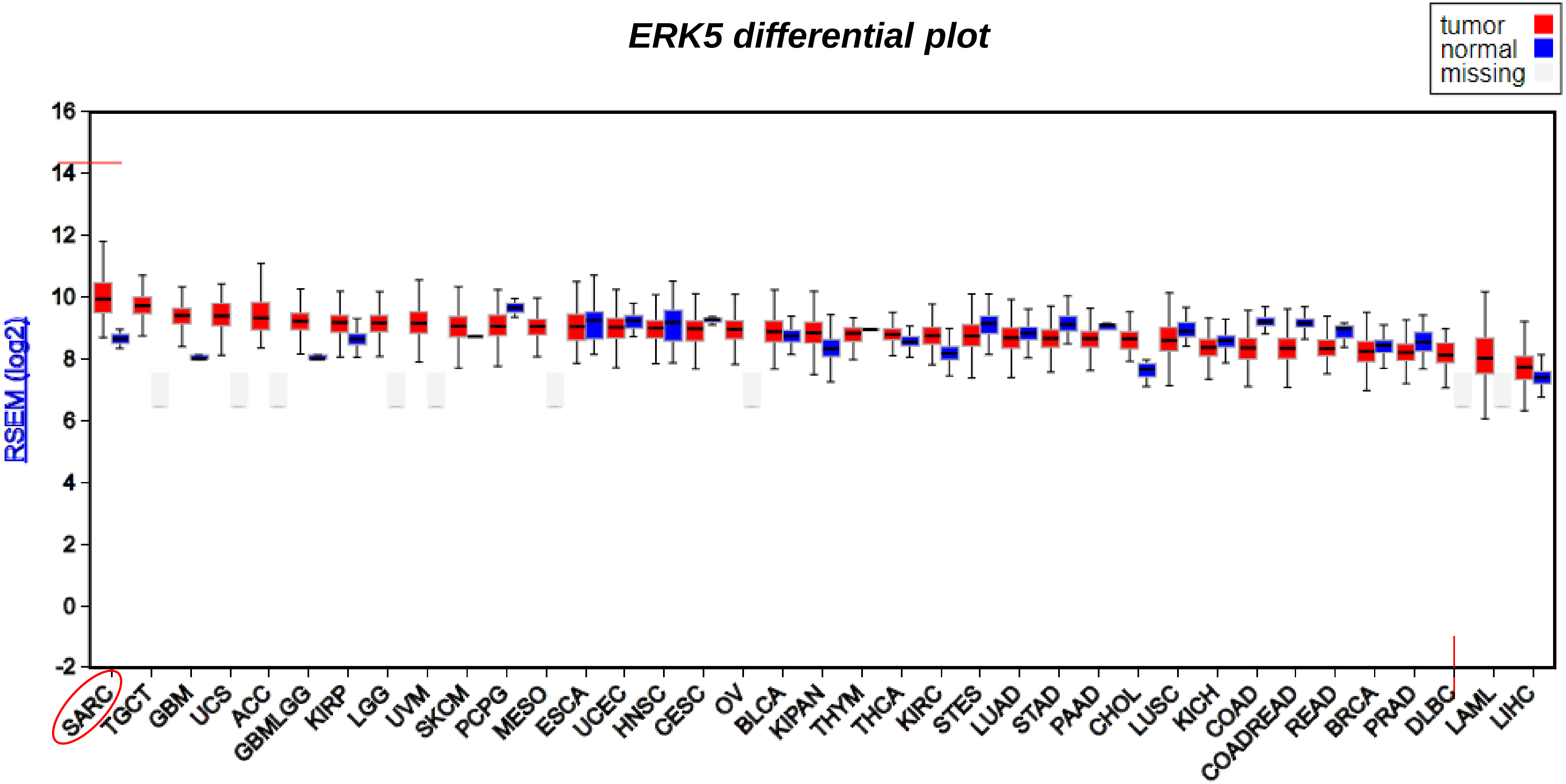

Supplementary Fig. 2

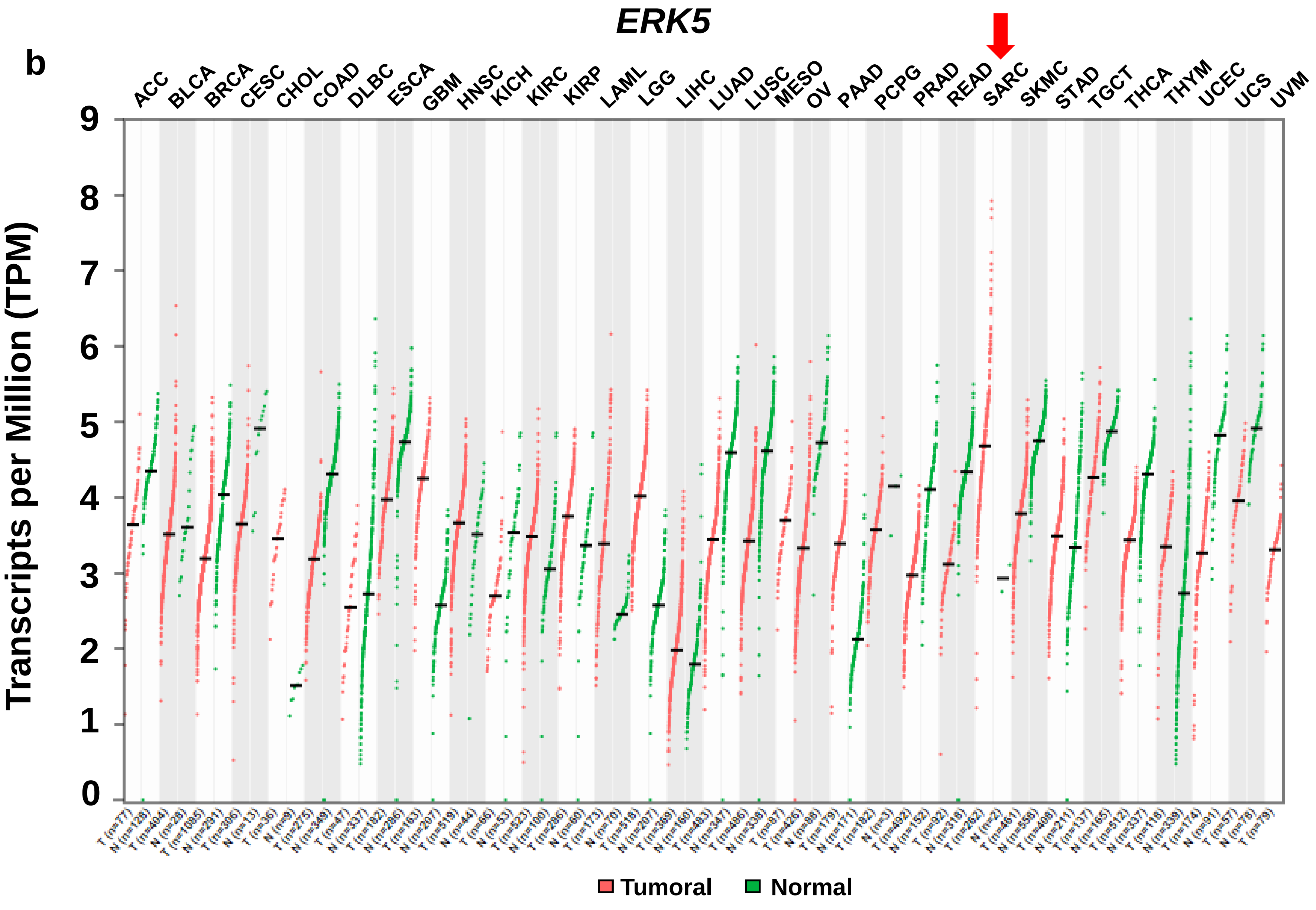

Supplementary Fig. 2

Supplementary Fig. 2. (a) *ERK5* mRNA expression levels across several tumor types including both tumoral and non-tumoral patients samples. Gene Expression Viewer tool available in Firebrowse was used as indicated in Material and Methods section. **(b)** Similar analyses were performed on the Gene Expression Profiling Interactive Analysis (GEPIA2) database. *ERK5* gene expression profile was quantified as Transcripts Per Million (TPM) and represented across all tumor samples and paired normal tissues.

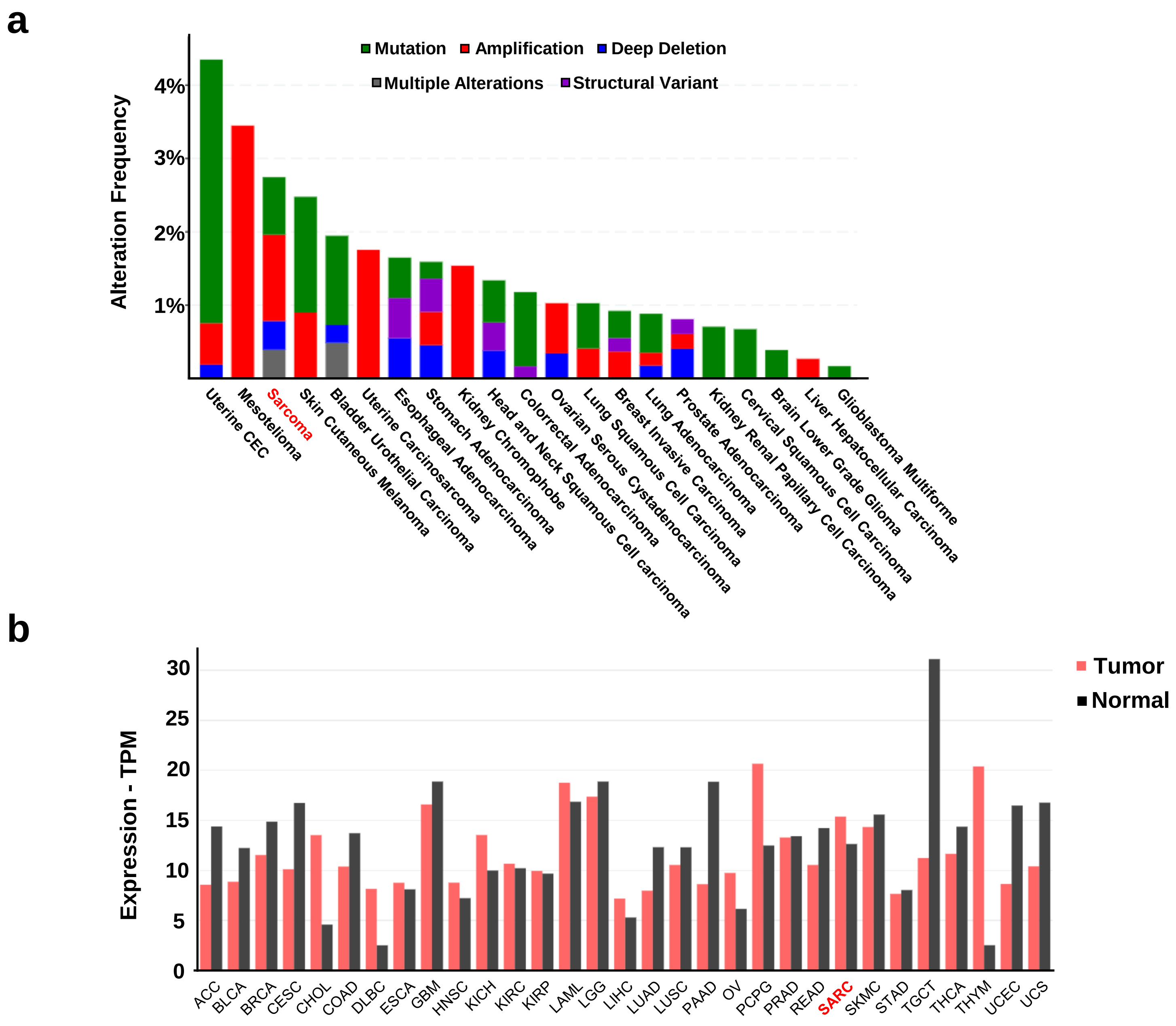

Supplementary Fig. 3

Supplementary Fig. 3. (a) Molecular alterations of *MEK5* in different tumor types using the complete list of TCGA PanCancer Atlas (N=10953 patients from 32 studies) available at cBioportal**. (b)** *MEK5* gene expression profile (quantified as transcripts per million TMP) across tumor patients samples and paired normal tissues obtained from GEPIA2 database.

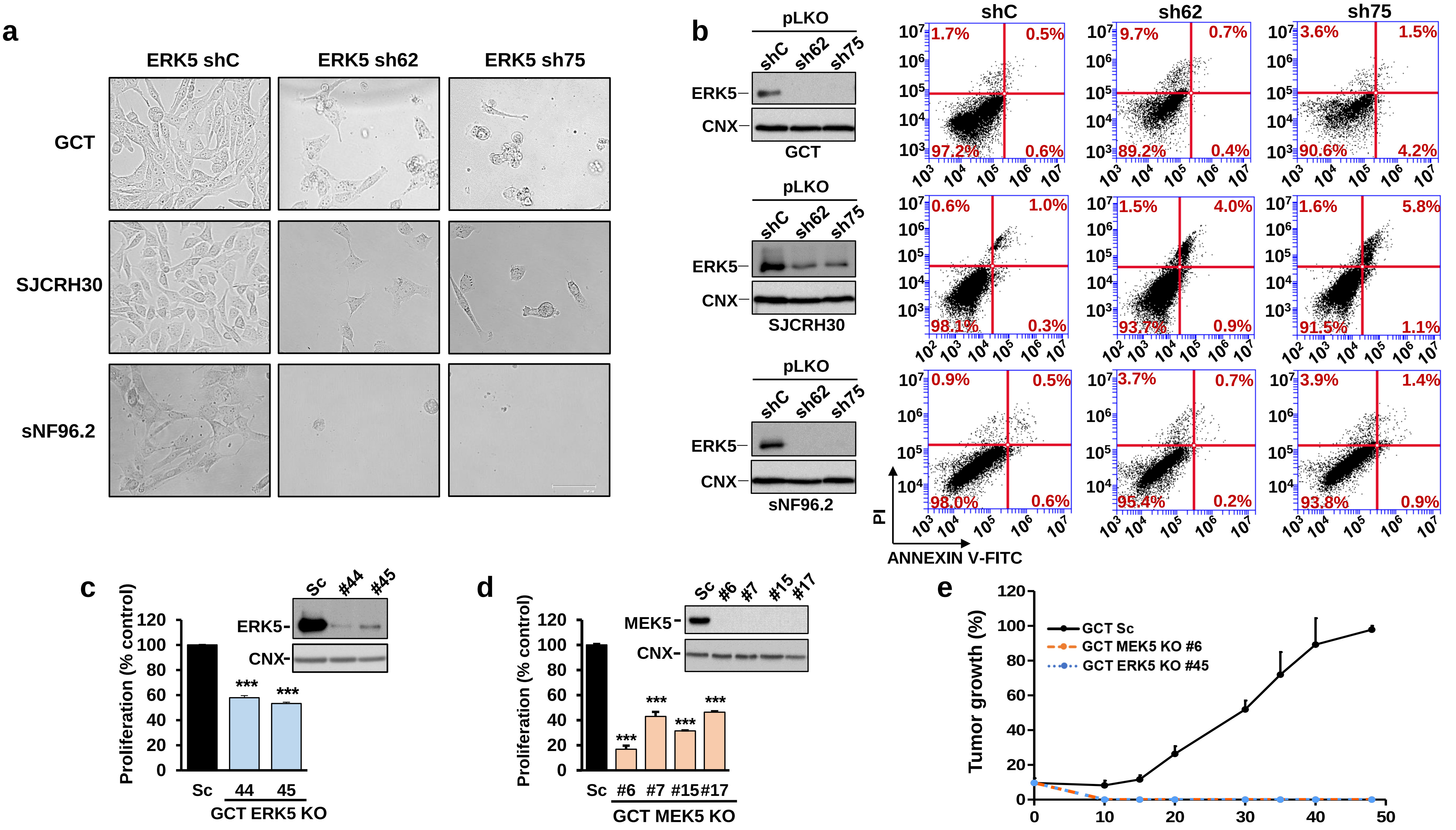

Supplementary Fig. 4

Supplementary Fig. 4. (a) Representative pictures of GCT, SJCRH30 and sNF96.2 sarcoma cell lines harboring short hairpin control (shC) or shERK5 RNA interfering sequences (sh62 and sh75). **(b)** Cells were cultured for 48 hours and apoptosis was analyzed by Annexin V-FITC assay. **(c)** GCT-ERK5 knockout clones #44 and #45. 1 mg of protein was immunoprecipitated and then probed with the C-terminal anti-ERK5 antibody. Calnexin was used as loading control. Proliferation was measured by the MTT assay at 5 days of culture. Data are represented as mean percentage from control GCT scramble cells ± SD of an experiment that was repeated twice. Statistical significance: ***, $p \leq 0.001$. **(d)** GCT MEK5 knockout clones #6, #7, #15 and #17. MEK5 lacking expression was corroborated by SDS-PAGE with 40 µg of protein extract followed by western blot with the anti-MEK5 antibody. Calnexin was used as loading control. Proliferation was performed by cell counting at 7 days of culture. Data are represented as mean percentage from control GCT scramble cells ± SD of an experiment that was repeated twice. Statistical significance: ***, $p \leq 0.001$. **(e)** GCT control Sc cells, GCT-ERK5 KO clone #45, and GCT-MEK5 KO clone #6 were xenografted subcutaneously in mice as indicated in Material and Methods section. Tumor growth was measured every five days and the experiment stopped after 48 days of implantation. GCT-ERK5 KO or -MEK5 KO clones were unable to develop tumors. Each point on the graph represents the mean tumor volume ± SEM and is plotted as a percentage of the volume achieved at the end point of the experiment.

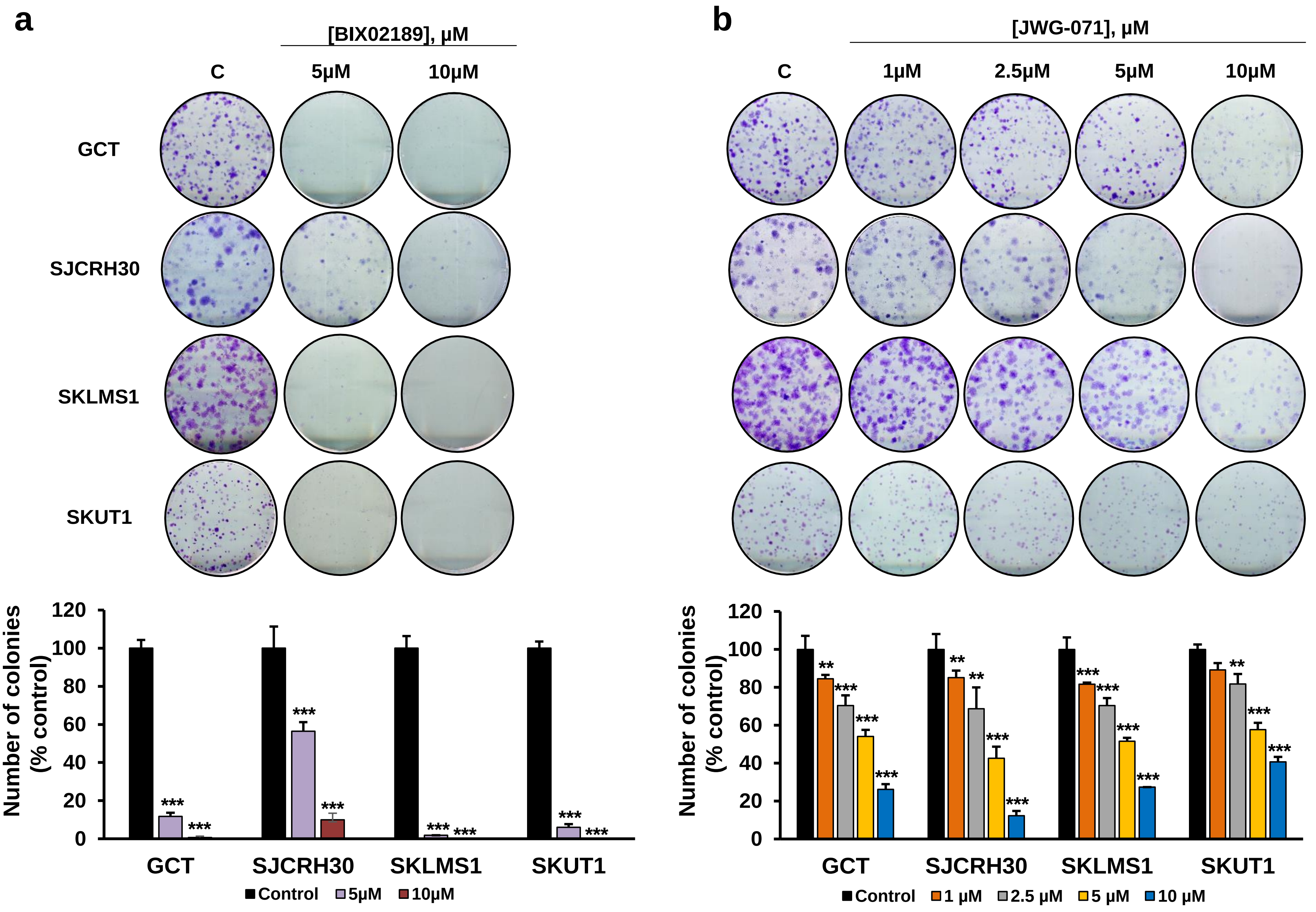

Supplementary Fig. 5

Supplementary Fig. 5. Sarcoma cells were plated in 6-well dishes and after 24 hours were treated with **(a)** BIX02189 or **(b)** JWG-071 at the indicated doses. Cells were cultured for 13 days, then stained with crystal violet, and colonies were counted by ImageJ Software. Results were relativized to control cells (untreated) and represented as mean ± SD of triplicates of an experiment that was repeated twice. Statistical significance: **, $p \leq 0.01$; ***, $p \leq 0.001$.

NOTE: the effect of the drug on the sNF96.2 cell line could not be analyzed by this procedure since these cells are not able to proliferate starting from a low number of plated cells as required by the assay.

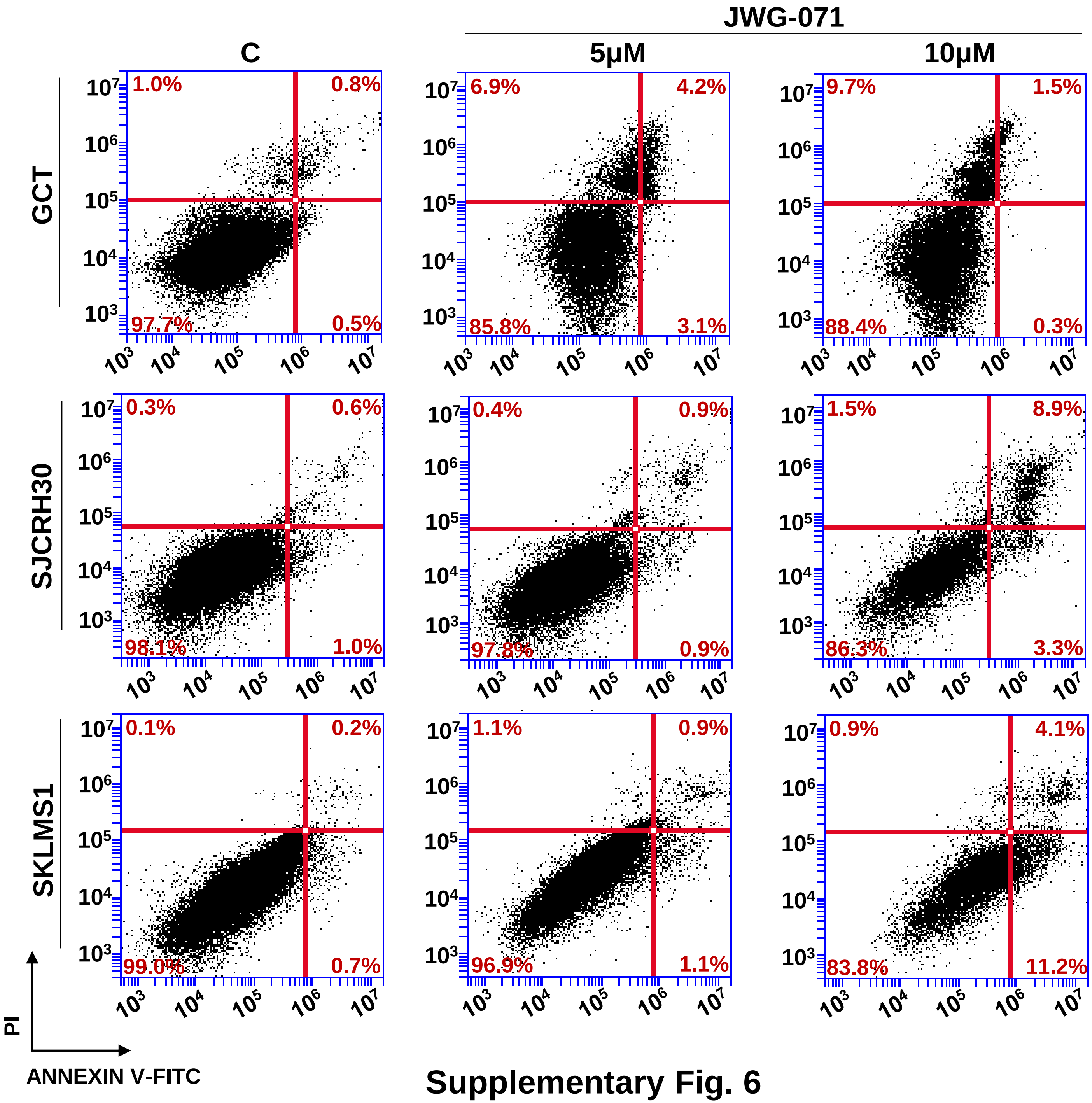

Supplementary Fig. 6

Supplementary Fig. 6. GCT, SJCRH30 and SKLMS1 cells, were either untreated (C) or treated with JWG-071 for 48 hours and apoptosis was analyzed by Annexin V-FITC assay.
